# ALG-2 activates the MVB sorting function of ALIX through relieving its intramolecular interaction

**DOI:** 10.1038/celldisc.2015.18

**Published:** 2015-07-21

**Authors:** Sheng Sun, Xi Zhou, Joe Corvera, Gary E Gallick, Sue-Hwa Lin, Jian Kuang

**Affiliations:** 1 Department of Experimental Therapeutics, The University of Texas MD Anderson Cancer Center, Houston, TX, USA; 2 The University of Texas Graduate School of Biomedical Sciences, Houston, TX, USA; 3 A&G Pharmaceuticals, Inc., Baltimore, MD, USA; 4 Department of Genitourinary Medical Oncology, The University of Texas MD Anderson Cancer Center, Houston, TX, USA; 5 Department of Molecular Pathology, The University of Texas MD Anderson Cancer Center, Houston, TX, USA

**Keywords:** ALIX, ALG-2, MVB sorting, EGFR, CHMP4

## Abstract

The modular adaptor protein ALIX is critically involved in endosomal sorting complexes required for transport (ESCRT)-mediated multivesicular body (MVB) sorting of activated epidermal growth factor receptor (EGFR); however, ALIX contains a default intramolecular interaction that renders ALIX unable to perform this ESCRT function. The ALIX partner protein ALG-2 is a calcium-binding protein that belongs to the calmodulin superfamily. Prompted by a defined biological function of calmodulin, we determined the role of ALG-2 in regulating ALIX involvement in MVB sorting of activated EGFR. Our results show that calcium-dependent ALG-2 interaction with ALIX completely relieves the intramolecular interaction of ALIX and promotes CHMP4-dependent ALIX association with the membrane. EGFR activation induces increased ALG-2 interaction with ALIX, and this increased interaction is responsible for increased ALIX association with the membrane. Functionally, inhibition of ALIX activation by ALG-2 inhibits MVB sorting of activated EGFR as effectively as inhibition of ALIX interaction with CHMP4 does; however, inhibition of ALIX activation by ALG-2 does not affect cytokinetic abscission or equine infectious anemia virus (EIAV) budding. These findings indicate that calcium-dependent ALG-2 interaction with ALIX is specifically responsible for generating functional ALIX that supports MVB sorting of ubiquitinated membrane receptors.

## Introduction

EGF binding to epidermal growth factor receptor (EGFR) on the cell surface induces both the activation and endocytosis of EGFR. Endocytosis of activated EGFR does not stop the signaling function of the receptor; however, it poises the receptor for being sorted into the intraluminal vesicles of multivesicular body (MVB) (MVB sorting), which terminates the signaling function of activated EGFR before its eventual degradation [1]. MVB sorting of endocytosed EGFR is carried out by multiple ESCRT complexes and associated proteins. Activated EGFR is first ubiquitinated by Cbl [2, 3]. This allows activated EGFR to be recognized by ESCRT-0, ESCRT-I, and ESCRT-II. The recognized EGFR is then sorted into the intraluminal vesicles of MVBs through membrane invagination and scission, which is driven by ESCRT-III assembly and disassembly [4]. Previous studies have demonstrated that EGF stimulation increases both MVB biogenesis and inward vesiculation within EGFR-containing MVBs [5]. How activated EGFR positively regulates these processes is not fully understood.

ALIX [6], also termed AIP1 [7] or Hp95 [8], is a widely expressed modular adaptor protein that is critically involved in a variety of ESCRT-III-mediated membrane remodeling processes, including MVB sorting of activated EGFR [9]. ALIX involvement in all defined ESCRT-III-mediated membrane remodeling processes requires ALIX interaction with the ESCRT-III component CHMP4 [9–18]. However, the native form of ALIX contains a default intramolecular interaction that renders ALIX unable to interact with CHMP4 [19, 20]. This predicts that MVB sorting of activated EGFR requires a regulatory mechanism that relieves the intramolecular interaction of ALIX.

Apoptosis-linked gene-2 product (ALG-2) [21] is a Ca^2+^-binding protein that interacts with ALIX in a Ca^2+^-dependent manner [6, 7]. The ALG-2 binding site near the C terminus of ALIX (801–812) is outside the region (1–746) that forms the intramolecular interaction [20]. ALG-2 is able to interact with the native form of recombinant ALIX in a Ca^2+^-dependent manner [6, 7], indicating that ALG-2 interaction with ALIX is not sensitive to the intramolecular interaction of ALIX. Endocytosis of cell surface receptors results in a rapid increase of calcium concentration in the vicinity of endosomes [22, 23], suggesting that ALG-2 activation is linked to endocytosis. Moreover, ALG-2 belongs to the penta-EF-hand subfamily in the EF hand or calmodulin superfamily of calcium-binding proteins [24], and calmodulin is known to regulate the activity of targeted proteins through inducing conformational changes that relieve the intramolecular interaction [25]. Collectively, these factors suggest the possibility that Ca^2+^-dependent ALG-2 interaction with ALIX activates the MVB sorting function of ALIX.

In this study, we defined the role of ALG-2 in the regulation of the intramolecular interaction of ALIX and MVB sorting of activated EGFR. Here, we present evidence that Ca^2+^-dependent ALG-2 interaction with ALIX relieves the intramolecular interaction of ALIX and that this newly identified regulatory event is specifically responsible for generating functional ALIX that supports MVB sorting of activated EGFR.

## Results

### ALG-2 interaction with ALIX relieves the intramolecular interaction of ALIX

ALG-2 interacts with four consecutive PxY motifs in ALIX (PPYPTYPGYPGY at 802–813) (Supplementary Figure S1A) [26]. To determine the effect of ALG-2 interaction with ALIX on the intramolecular interaction of ALIX, we first verified the previous findings that GST-ALG-2 interacts with cytosolic ALIX in the presence of 10 μm calcium (Supplementary Figure S1B), and that the E47A/E114A mutant form of ALG-2 (Mut ALG-2), which is defective in calcium binding, does not interact with cytosolic ALIX in the presence of calcium (Supplementary Figure S1C) [26]. We then incubated cytosolic proteins from HEK293 cells with GST-ALG-2 and/or calcium and examined the effect of that incubation on the accessibility of the docking sites for Src and CHMP4 in the N-terminal Bro1 domain, the docking site for viral GAG proteins in the middle V domain, and the docking site for TSG101 in the proline-rich domain. We previously demonstrated that the 1A3 and 2H12 anti-ALIX monoclonal antibodies recognize the docking sites for Src and viral GAG proteins, respectively. In contrast, the 1A12 and 1F7 anti-ALIX antibodies recognize epitopes in ALIX that are insensitive to the intramolecular interaction [19, 27].

**Figure 1:**
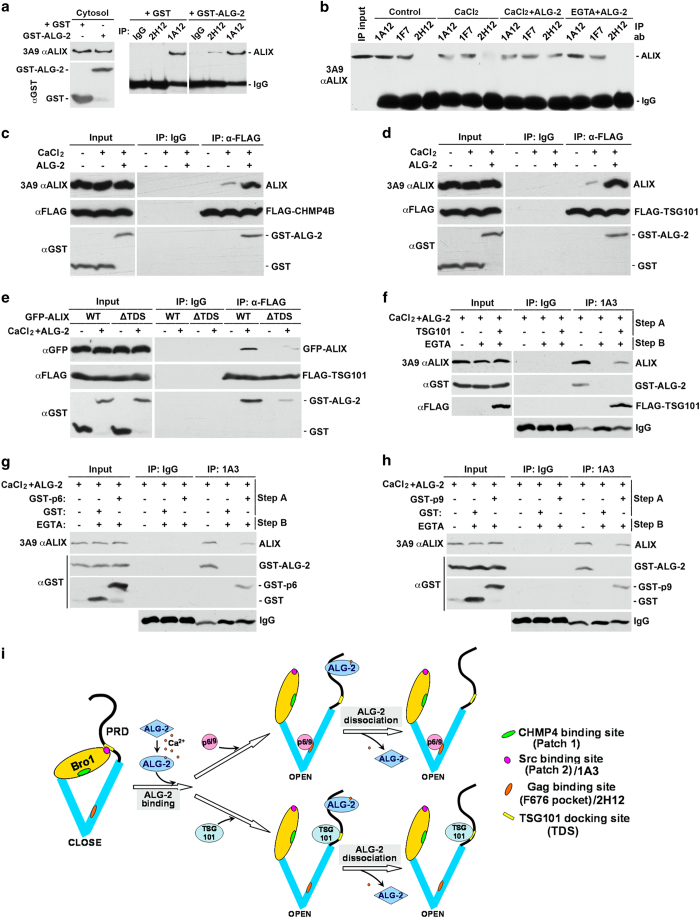
ALG-2 interaction with ALIX relieves the intramolecular interaction of ALIX. (**a**) Cytosolic proteins from HEK293 cells were mixed with GST or GST-ALG-2 and immunoprecipitated with mouse IgG or indicated monoclonal anti-ALIX antibodies. Input proteins and immunocomplexes were immunoblotted with indicated antibodies to visualize ALIX, IgG, GST, and GST-ALG-2. (**b**) Identical amounts of cytosolic proteins from HEK293 cells were mock treated (control) or mixed with CaCl_2_, CaCl_2_ plus GST-ALG-2 (CaCl_2_+ALG-2), or EGTA plus GST-ALG-2 (EGTA+ALG-2), and same aliquots from each sample were immunoprecipitated with each of the three indicated monoclonal anti-ALIX antibodies. Input proteins and immunocomplexes were immunoblotted to visualize ALIX and IgG. (**c**, **d**) Cytosolic proteins prepared from HEK293 cells ectopically expressing FLAG-CHMP4B (**c**) or FLAG-TSG101 (**d**) were mixed with CaCl_2_ or CaCl_2_ plus GST-ALG-2, and samples were immunoprecipitated with a monoclonal anti-FLAG antibody. Input proteins and immunocomplexes were immunoblotted with indicated antibodies to visualize ALIX, GST, GST-ALG-2, and FLAG-CHMP4B (**c**) or FLAG-TSG101 (**d**). (**e**) Cytosolic proteins prepared from HEK293 cells ectopically co-expressing FLAG-TSG101 and WT GFP-ALIX or FLAG-TSG101 and ∆TDS GFP-ALIX were immunoprecipitated with polyclonal anti-FLAG antibodies in the presence or absence of supplemented GST-ALG-2 and CaCl_2_. Input proteins and immunocomplexes were immunoblotted with indicated antibodies to visualize GFP-ALIX, FLAG-TSG101, GST, and GST-ALG-2. (**f**) Cytosolic proteins prepared from HEK293 cells with or without expression of FLAG-TSG101 were first incubated with GST-ALG-2 plus CaCl_2_ for 30 min (step A), and then incubated for another 30 min in the presence or absence of added EGTA (step B). After samples were immunoprecipitated with the 1A3 antibody, input proteins and immunocomplexes were immunoblotted with indicated antibodies to visualize ALIX, GST-ALG-2, and FLAG-TSG101. (**g**, **h**) Cytosolic proteins prepared from HEK293 cells were mixed with GST or GST-p6 in **g** and GST or GST-p9 in **h**. Samples were then processed as described in **f**, except that input proteins and immunocomplexes were immunoblotted with indicated antibodies to visualize ALIX, GST-ALG-2, or GST/GST-p6 (**g**) or GST/GST-p9 (**h**). (**i**) A schematic illustration of ALG-2-induced relief of the intramolecular interaction of ALIX and the role of partner protein binding in maintenance of the open conformation of ALIX after ALG-2 dissociation. ALG-2, apoptosis-linked gene-2 product; GST, glutathione *S*-transferase; IgG, immunoglobulin G.

We first added glutathione *S-*transferase (GST) or GST-ALG-2 alone to the cytosolic fraction of HEK293 cells and immunoprecipitated each sample with the 2H12 or 1A12 antibody. As the cytosolic fraction is likely to have background levels of calcium, a small portion of added GST-ALG-2 may interact with cytosolic ALIX. As shown in Figure 1a, the GST-ALG-2, but not GST, addition induced a low level of ALIX immunoprecipitation by the 2H12 antibody, whereas neither GST nor GST-ALG-2 addition changed ALIX immunoprecipitation by the 1A12 antibody. These results suggest that ALG-2 interaction with ALIX relieves the intramolecular interaction of ALIX. To test this possibility, we then added nothing, CaCl_2_, GST-ALG-2 plus CaCl_2_ or GST-ALG-2 plus 5 mm EGTA to the cytosolic fraction of HEK293 cells and immunoprecipitated each of the four samples with the 1A12, 1F7 or 2H12 antibody. As shown in Figure 1b, both the 1A12 and 1F7 antibodies immunoprecipitated high levels of ALIX from the four samples. In contrast, the 2H12 antibody did not immunoprecipitate detectable levels of ALIX from control cytosol or cytosol supplemented with GST-ALG-2 plus EGTA. However, the 2H12 antibody immunoprecipitated low detectable levels of ALIX from cytosol with CaCl_2_ added, in which a low level of endogenous ALG-2 could be activated, and immunoprecipitated high levels of ALIX from cytosol to which both CaCl_2_ and GST-ALG-2 had been added. Consistent with these results, the 1A3 antibody also immunoprecipitated undetectable levels of ALIX from control cytosol, barely detectable levels of ALIX from cytosol with CaCl_2_ added, and readily detectable levels of ALIX from cytosol supplemented with both CaCl_2_ and GST-ALG-2 (Supplementary Figure S1D). Testing ALIX interaction with partner proteins showed that cytosolic ALIX did not co-immunoprecipitate with ectopically expressed FLAG-CHMP4B or FLAG-TSG101 in control cytosol. The addition of CaCl_2_ plus GST-ALG-2 to the cytosol promoted ALIX co-immunoprecipitation with FLAG-CHMP4B and FLAG-TSG101 more markedly than the addition of CaCl_2_ alone (Figures 1c and d). Collectively, these results demonstrate that Ca^2+^-dependent ALG-2 interaction with cytosolic ALIX completely relieves the intramolecular interaction of ALIX.

ALG-2 has been reported to interact with both ALIX and TSG101 in a calcium-dependent manner [28], raising the possibility that ALG-2-induced ALIX interaction with TSG101 is an indirect event requiring direct interaction of ALG-2 with both ALIX and TSG101. To explore this possibility, we deleted the TSG101 docking site in green fluorescent protein (GFP)-ALIX and determined whether GST-ALG-2 plus CaCl_2_ still promotes its interaction with ALIX. As shown in Figure 1e, deletion of the TSG101 docking site markedly reduced the ability of GFP-ALIX to interact with FLAG-TSG101 even in the presence of supplemented GST-ALG-2 plus CaCl_2_. We also treated the FLAG-TSG101/ALIX/GST-ALG-2 immunocomplex immobilized on anti-FLAG antibody affinity beads with 5 mm EGTA to release GST-ALG-2 and determined whether ALIX interaction with immunoprecipitated FLAG-TSG101 remains. Although the EGTA treatment quantitatively dissociated GST-ALG-2 from the immunocomplex, it only released a low level of ALIX (Supplementary Figure S1E). Together, these results demonstrate that ALG-2-induced ALIX interaction with TSG101 is largely a direct event.

To determine whether ALG-2-induced conformational change in ALIX is a reversible event, we induced the open conformation of ALIX by incubating cytosolic proteins from HEK293 cells with GST-ALG-2 plus CaCl_2_ and then dissociated GST-ALG-2 from ALIX by chelating calcium with EGTA. Immunoprecipitation of end products with the conformation-sensitive 1A3 antibody showed that EGTA treatment reversed the ALG-2-induced open conformation of ALIX (Figures 1f and h). However, when cytosolic proteins contained ectopically expressed FLAG-TSG101 (Figure 1f), or were mixed with GST-tagged HIV GAG protein p6 (Figure 1g) or EIAV GAG protein p9 (Figure 1h), EGTA treatment neither reversed the ALG-2-induced immunoprecipitation of ALIX by the 1A3 antibody nor ALIX interaction with these binding partners. These results demonstrate that ALG-2-induced ALIX interaction with partner proteins maintains the open conformation of ALIX, even after ALG-2 dissociation.

Collectively, the above results support the model illustrated in Figure 1h, in which Ca^2+^-dependent ALG-2 interaction with the proline-rich domain of ALIX induces a global conformational change in ALIX that relieves the intramolecular interaction of ALIX and in which the open conformation of ALIX can be maintained after ALG-2 dissociation by newly recruited partner proteins.

### ALG-2 interaction with ALIX has a rate-limiting role in CHMP4-dependent ALIX association with the membrane

**Figure 2:**
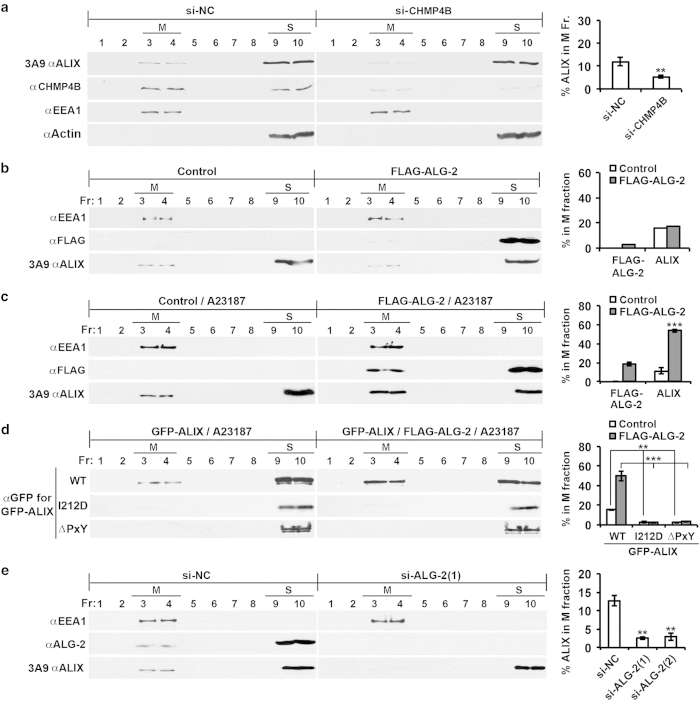
ALG-2 interaction with ALIX has a rate-limiting role in CHMP4-dependent ALIX association with the membrane. (**a**) HEK293 cells were transfected with negative-control siRNA (si-NC) or two CHMP4B-specific siRNAs (si-CHMP4B), and the PNSs from these cells were fractionated by membrane flotation centrifugation. Same volumes of aliquots were taken and immunoblotted with indicated antibodies; M and S protein fractions are indicated (left panel). The average percentages of ALIX in the M fraction and s.d. were determined for each cell condition from three independent experiments and plotted (right panel). (**b**) HEK293 cells were transfected with an empty vector (control) or a FLAG-ALG-2 expression vector (FLAG-ALG-2), and processed as described in **a** (left panel). The percentages of FLAG-ALG-2 and ALIX in the M fraction were determined and plotted (right panel). (**c**) HEK293 cells were processed as described for **b**, except that cells were treated with the calcium ionophore A23187 for 15 min before cell extraction (left panel). The average percentages of ALIX and FLAG-ALG-2 in the M fraction and s.d. were determined from three independent experiments and plotted (right panel). (**d**) HEK293 cells ectopically expressing indicated forms of GFP-ALIX and FLAG-ALG-2 were processed as described for **c** (left panel). The average percentages of GFP-ALIX in the M fraction and s.d. were determined from three independent experiments and plotted (right panel). (**e**) HEK293 cells were transfected with indicated siRNAs and processed as described for **a** (left panel). The average percentages of ALIX in the M fraction and s.d. were determined from three independent experiments and plotted (right panel). ALG-2, apoptosis-linked gene-2 product; GFP, green fluorescent protein; M, membrane fraction; PNS, post-nuclear supernatant; S, soluble fraction; si-NC, negative-control small interfering RNA; siRNA, small interfering RNA.

Inducing the open conformation of ALIX by deleting or occupying one of the intramolecular interaction sites in ALIX has been demonstrated to increase membrane-associated ALIX from ~10 to ~50% [20]. To determine whether inducing the open conformation of ALIX by ALG-2 binding also promotes ALIX association with the membrane, we first tested our previous finding that ALIX association with the membrane in HEK293 cells is mainly achieved through ALIX interaction with membrane-bound CHMP4 [9]. HEK293 cells were transfected with a negative-control small interfering RNA (siRNA) (si-NC) or two CHMP4B-specific siRNAs (si-CHMP4B), and the effect on ALIX association with the membrane was determined using a method referred to as membrane flotation centrifugation. This method was established in previous studies to separate all membrane vesicles from soluble proteins [29, 30]. We used early-endosome antigen 1 (EEA1) and β-actin as internal markers for membrane (M) fractions and soluble protein (S) fractions, respectively. As shown in Figure 2a, partial CHMP4B knockdown (~70%) decreased the membrane-associated ALIX from ~12 to ~5%, supporting our previous finding.

Next, we ectopically expressed FLAG-ALG-2 in HEK293 cells and examined the effect of that expression on ALIX association with the membrane. Although the FLAG-ALG-2 expression alone produced little effect on the percentage of membrane-associated ALIX (Figure 2b), the FLAG-ALG-2 expression in conjunction with a brief cell treatment with the calcium ionophore A23187 increased the percentage of membrane-associated ALIX from ~11 to ~53% (Figure 2c). Consistent with the requirement of a calcium ionophore for FLAG-ALG-2 to promote ALIX association with the membrane, expression of Mut FLAG-ALG-2 did not increase the percentage of membrane-associated ALIX, even when cells were treated with A23187 (Supplementary Figure S2A). These results demonstrate that ALG-2 interaction with ALIX promotes ALIX association with the membrane. As further proof of this concept, incubation of the post-nuclear supernatant (PNS) of HEK293 cell lysates with GST-ALG-2 plus CaCl_2_, but not GST plus CaCl_2_, increased membrane-associated ALIX from ~14 to ~55% (Supplementary Figure S2B).

Further, we ectopically expressed wild-type (WT), CHMP4-binding site-mutated (I212D) [13, 15] or ALG-2-binding site (amino acids 800–814)-deleted (∆PxY) [26] GFP-ALIX in HEK293 cells with or without co-expression of FLAG-ALG-2 and examined the association of each with the membrane after A23187 treatment. As shown in Figure 2d and Supplementary Figure S2C, the FLAG-ALG-2 co-expression increased the membrane association of WT GFP-ALIX similarly as it did to the endogenous ALIX. However, neither ΔPxY GFP-ALIX nor I212D GFP-ALIX was readily detectable in the membrane fractions irrespective of the FLAG-ALG-2 expression. These results demonstrate that both the basal level of ALIX association with the membrane and ALG-2-induced high levels of ALIX association with the membrane are largely achieved through ALIX interaction with the membrane-bound CHMP4. As further proof of this concept, the addition of GST-ALG-2 plus CaCl_2_ to the PNS of HEK293 cells ectopically expressing WT GFP-ALIX or I212D GFP-ALIX increased the membrane association of WT GFP-ALIX but not of I212D GFP-ALIX (Supplementary Figure S2D).

To characterize the physiological role of ALG-2 in ALIX association with the membrane, we transfected HEK293 cells with a negative-control siRNA (si-NC) or one of the two ALG-2-specific siRNAs, designated si-ALG-2(1) and si-ALG-2(2), and determined the effect of ALG-2 knockdown on ALIX association with the membrane. As shown in Figure 2e and Supplementary Figure S2E, ALG-2 knockdown by either of si-ALG-2(1) or si-ALG-2(2) decreased membrane-associated ALIX from ~13 to 2–3%, indicating that ALG-2 has a physiologically important role in CHMP4-dependent ALIX association with the membrane.

### Increased ALG-2 interaction with ALIX is responsible for increased ALIX association with the membrane in EGF-stimulated cells

**Figure 3:**
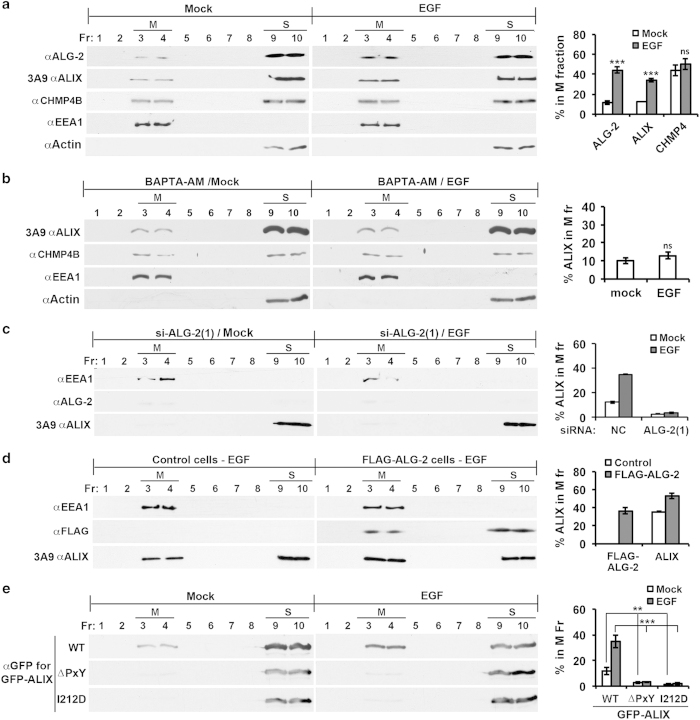
Increased ALG-2 interaction with ALIX is responsible for increased ALIX association with the membrane in EGF-stimulated cells. (**a**) HEK293 cells were mock treated or stimulated with EGF for 1 h and processed as described in Figure 2a (left panel). The average percentages of ALIX, ALG-2, and CHMP4B in the M fraction and s.d. were determined from three independent experiments and plotted (right panel). (**b**) HEK293 cells were processed as described for **a**, except that cells were treated with BAPTA-AM starting 1 h before EGF stimulation (left panel). The average percentages of ALIX in the M fraction and s.d. were determined from three independent experiments and plotted (right panel). (**c**) Serum-starved HEK293 cells transfected with indicated siRNAs were mock treated or stimulated with EGF for 1 h and processed as described in Figure 2a. The average percentages of ALIX and ALG-2 in the M fraction were determined from two independent experiments, and error bars indicate the range of the results (right panel). (**d**) Serum-starved HEK293 cells transfected with an empty vector (control) or a FLAG-ALG-2 expression vector were stimulated with EGF for 1 h and processed as described in Figure 2a. The percentages of ALIX in the M fraction were determined from two independent experiments, and error bars indicate the range of the results (right panel). (**e**) HEK293 cells ectopically expressing indicated forms of GFP-ALIX were mock treated or stimulated with EGF for 1 h and then processed as described in Figure 2a (left panel). The percentages of GFP-ALIX in the M fraction and s.d. were determined from three independent experiments and plotted (right panel). ALG-2, apoptosis-linked gene-2 product; EGF, epidermal growth factor; GFP, green fluorescent protein; M, membrane fraction; NS, not significant; siRNA, small interfering RNA.

Our recent results demonstrate that EGF stimulation of HEK293 cells induces a threefold increase in CHMP4-dependent ALIX association with the membrane [9]. To determine the role of ALG-2 in the EGF-induced increase in ALIX association with the membrane, we first determined the effect of EGF stimulation on ALG-2 interaction with ALIX in 0.1% Triton X-100-solubilized cell lysates by co-immunoprecipitation. Under the same concentration of added CaCl_2_ (10 μm), 3.5-fold more ALG-2 interacted with ALIX in EGF-stimulated cells than in control cells (Supplementary Figure S3A). We then determined the effect of EGF stimulation on ALG-2 association with the membrane using membrane flotation centrifugation. As shown in Figure 3a, EGF stimulation of HEK293 cells not only increased membrane-associated ALIX from ~12 to ~35%, as previously observed [9], but also increased membrane-associated ALG-2 from ~11 to ~44%. In contrast, CHMP4B was evenly distributed between the membrane and soluble protein fractions irrespective of EGF stimulation. Further, we determined the effect of the cell-permeable Ca^2+^ chelator BAPTA-AM on EGF-induced increase in ALIX association with the membrane. As shown in Figure 3b, BAPTA-AM treatment starting 1 h before EGF stimulation prevented EGF-induced increase in ALIX association with the membrane. Together, these results generated a hypothesis that increased ALG-2 interaction with ALIX is responsible for increased ALIX association with the membrane in EGF-stimulated cells.

To test this hypothesis, we determined the effect of ALG-2 knockdown or overexpression on EGF-induced ALIX association with the membrane. Irrespective of EGF stimulation, ALG-2 knockdown reduced the level of membrane-associated ALIX to barely detectable levels (Figure 3c; Supplementary Figure S3B). In contrast, ectopic expression of WT FLAG-ALG-2, but not Mut FLAG-ALG-2, increased the membrane-associated ALIX in EGF-stimulated cells from ~35 to ~53% (Figure 3d; Supplementary Figure S3C). Moreover, we ectopically expressed WT GFP-ALIX, ΔPxY GFP-ALIX or I212D GFP-ALIX in HEK293 cells and determined their membrane association in control cells and EGF-stimulated cells. As shown in Figure 3e, EGF stimulation increased the percentage of membrane-associated WT GFP-ALIX similarly as it did to that of endogenous ALIX. However, both the ALG-2 non-interactive ΔPxY GFP-ALIX and the CHMP4 non-interactive I212D GFP-ALIX were barely detectable in the membrane fraction, irrespective of EGF stimulation. These results demonstrate that EGF stimulation leads to increased ALG-2 interaction with ALIX and that this increased interaction is responsible for increased ALIX association with the membrane.

### ALG-2-unlocked ALIX quantitatively associates with the membrane

**Figure 4:**
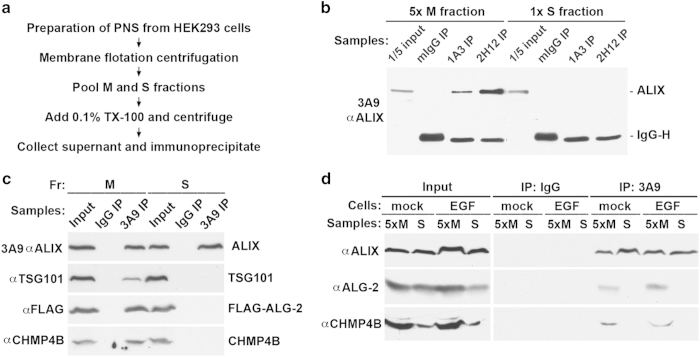
ALG-2-unlocked ALIX quantitatively associates with the membrane. (**a**) An experimental flowchart for determination of the distribution of ALG-2-unlocked ALIX between the M and S fractions by immunoprecipitation. (**b**) After HEK293 cells were processed as described in **a**, 5:1 volumes of aliquots taken from the pooled M and S fractions were immunoprecipitated with mouse IgG or indicated anti-ALIX antibodies. Immunoprecipitation input and immunocomplexes were immunoblotted with the 3A9 antibody to visualize ALIX and IgG. (**c**) HEK293 cells were transfected with FLAG-ALG-2 and treated with A23187 for 15 min. Cells were processed as described in **a**, and equal volumes of aliquots taken from the pooled M fraction and S fractions were immunoprecipitated with the 3A9 antibody. Immunoprecipitation input and immunocomplexes were immunoblotted with indicated antibodies to visualize ALIX, FLAG-ALG-2, TSG101, and CHMP4B. (**d**) Serum-starved HEK293 cells were mock treated or stimulated with EGF for 1 h, and both serum-starved cells and EGF-stimulated cells were processed as described in **a**. From the pooled M and S fractions, 5:1 volumes of aliquots were taken and immunoprecipitated with mouse IgG or the 3A9 anti-ALIX antibody. Immunoprecipitation inputs and immunocomplexes were immunoblotted with indicated antibodies to visualize ALIX, ALG-2, and CHMP4B. ALG-2, apoptosis-linked gene-2 product; EGF, epidermal growth factor; IgG, immunoglobulin G; M, membrane fraction; PNS, post-nuclear supernatant; S, soluble fraction.

ALIX is a versatile protein that is also involved in actin-based cytoskeleton assembly [31, 32] and integrin-mediated cell adhesion [33, 34]. Since ALIX involvement in these non-ESCRT processes does not seem to require an open conformation of ALIX, an interesting question is whether ALG-2-unlocked ALIX can perform both ESCRT and non-ESCRT functions. To answer this question, we determined the distribution of ALG-2-unlocked ALIX in control HEK293 cells, ALG-2-overexpressing HEK293 cells, and EGF-stimulated HEK293 cells using the experimental strategy diagrammed in Figure 4a.

The PNS from regular HEK293 cells was fractionated by membrane flotation centrifugation. After pooled membrane (M) fractions and soluble (S) fractions were adjusted to contain 0.1% Triton X-100 and comparable levels of ALIX, soluble proteins were immunoprecipitated with the conformation-sensitive 1A3 and 2H12 antibodies. As observed in our previous studies [19, 27], both antibodies specifically immunoprecipitated ALIX from the M fraction (Figure 4b), indicating that the basal level of ALG-2-unlocked ALIX in control cells quantitatively associates with the membrane.

The PNS from A23187-treated HEK293 cells ectopically expressing FLAG-ALG-2 was next fractionated by membrane flotation centrifugation, and proteins soluble in 0.1% Triton X-100 were prepared from the pooled M fractions and S fractions. After samples were immunoprecipitated with the conformation-insensitive 3A9 antibody, immunocomplexes were immunoblotted to visualize co-immunoprecipitation of FLAG-ALG-2, TSG101, and CHMP4B with ALIX. Although the immunocomplexes from the M fraction contained high levels of all three of the proteins examined, the immunocomplexes from the S fraction did not contain detectable levels of any of the three proteins (Figure 4c). These results indicate that ALG-2-induced high levels of unlocked ALIX quantitatively interact with membrane-bound CHMP4.

Further, the PNSs from serum-starved and EGF-stimulated cells were fractionated by membrane flotation centrifugation, and the co-immunoprecipitation strategy was again used to examine ALIX interaction with endogenous ALG-2 and CHMP4 in the M and S fractions. Under both cellular conditions, readily detectable levels of ALIX interaction with ALG-2 and CHMP4B were again observed only in the M fraction (Figure 4d). These results indicate that EGF-induced high levels of unlocked ALIX also quantitatively interact with membrane-bound CHMP4.

### Unlocking ALIX by ALG-2 has a rate-limiting role in MVB sorting of activated EGFR

The critical role of ALG-2 interaction with ALIX in ALIX association with the membrane predicts that ALG-2 has a rate-limiting role in MVB sorting of activated EGFR. To test this prediction, we measured the effect of ALG-2 knockdown on MVB sorting of activated EGFR by the proteinase K protection assay, which measures the percentage of cytoplasmic EGFR that cannot be digested by proteinase K in the absence of the membrane-solubilizing detergent, Triton X-100. As shown in our recent studies [9], EGF stimulation of HEK293 cells for 30 min increased the percentage of the proteinase K-insensitive EGFR from ~5 to ~60% (Supplementary Figure S4).

**Figure 5:**
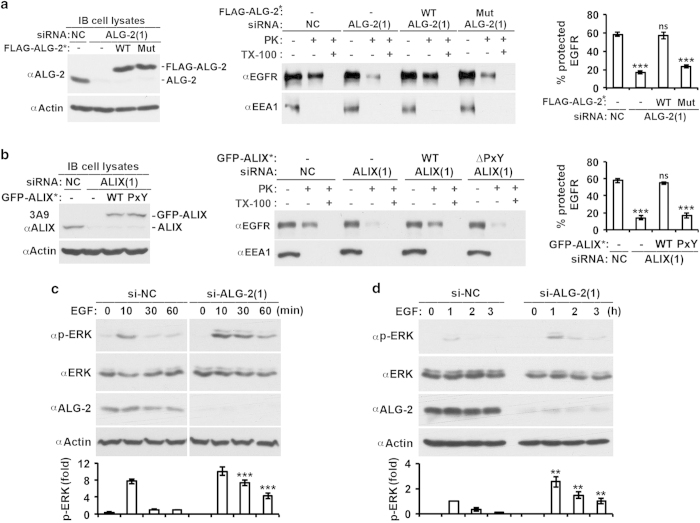
Activation of ALIX by ALG-2 has a rate-limiting role in MVB sorting of activated EGFR. (**a**) HEK293 cells were first transfected with si-NC or si-ALG-2(1) and cultured for 48 h. These cells were then transfected with the expression plasmids for WT FLAG-ALG-2* or Mut FLAG-ALG-2* (E47A/E114A mutation in ALG-2) and cultured for another 12 h. After serum starvation for 12 h, these cells were stimulated with EGF for 30 min and assayed for MVB sorting of activated EGFR by the proteinase K protection assay (left and middle panels). The average percentages of protected EGFR and s.d. were determined from three independent experiments and plotted (right panel). (**b**) HEK293 cells were transfected with indicated siRNAs and expression plasmids for GFP-ALIX* and processed as described in **a**. (**c, d**) HEK293 cells transfected with indicated siRNAs were stimulated with EGF for the indicated minutes (**c**) or hours (**d**), and cell lysates were immunoblotted with indicated antibodies to visualize phosphorylated ERK1/2 (p-ERK1/2), ERK1/2, and actin (top panel). The relative levels of p-ERK at different time points were determined, normalized against the level at 60 min in **c** and 1 h in **d**. The average levels of p-ERK and s.d. were determined from three independent experiments and plotted (bottom panel). ALG-2, apoptosis-linked gene-2 product; EGFR, epidermal growth factor receptor; GFP, green fluorescent protein; MVB, multivesicular body; NS, not significant; si-NC, negative-control small interfering RNA; siRNA, small interfering RNA.

HEK293 cells were transfected with si-ALG-2(1) with or without co-transfection of WT or Mut FLAG-ALG-2, which was ALG-2-siRNA(1) insensitive (FLAG-ALG-2*). Immunoblots of cell lysates showed that the si-ALG-2(1) transfection reduced ALG-2 expression by >90%, and that WT and Mut FLAG-ALG-2* were expressed at levels similar to the original level of endogenous ALG-2 (Figure 5a, left panel). The proteinase K protection assay showed that ALG-2 knockdown reduced the percentage of the proteinase K-insensitive EGFR from ~60 to ~17%. Although the inhibitory effect of ALG-2 knockdown was fully rescued to the original level of ~60% by the ectopic expression of WT FLAG-ALG-2*, it was slightly rescued to 24% by the expression of Mut FLAG-ALG-2* (Figure 5a, middle and right panels). The slight rescuing effect of Mut FLAG-ALG-2* could be due to its residual ability to activate the MVB sorting function of ALIX or non-ALIX-related functions of the mutant protein. In any event, these results demonstrate that ALG-2 interaction with ALIX has a major role in generating functional ALIX that supports MVB sorting of activated EGFR.

To further test this conclusion, we measured the effect of the ∆PxY mutation on the ability of ALIX to support MVB sorting of activated EGFR. HEK293 cells were transfected with si-ALIX(1) with or without co-transfection of WT GFP-ALIX or ∆PxY GFP-ALIX, which was si-ALIX(1) insensitive (GFP-ALIX*). Immunoblots of cell lysates showed that the si-ALIX(1) transfection reduced ALIX expression by >90%, and that the level of GFP-ALIX* expression was similar to that of the original endogenous ALIX (Figure 5b, left panel). The proteinase K protection assay showed that ALIX knockdown reduced the percentage of proteinase K-insensitive EGFR from ~60 to ~14%. Although the inhibitory effect of ALIX knockdown was fully rescued by the ectopic expression of WT GFP-ALIX*, it was not rescued by the expression of ∆PxY GFP-ALIX* (Figure 5b, middle and right panels). These results support our conclusion.

The ALIX-supported MVB sorting has been demonstrated to be a critical determinant in the highly transient nature (10 min) of EGF-induced ERK1/2 activation [9], which reflects rapid silencing of activated EGFR before its degradation. As the MVB supporting function of ALIX requires its activation by ALG-2, ALG-2 knockdown should result in sustained activation of ERK1/2. To test this prediction, we determined the effect of ALG-2 knockdown on the kinetics of ERK activation during the first 60 min of EGF stimulation and from 1 to 3 h afterward. As observed with the effect of ALIX knockdown [9], ALG-2 knockdown promoted sustained activation of ERK1/2 both in the first 60 min of stimulation (Figure 5c) and from 1 to 3 h of stimulation (Figure 5d). These results lend further credence to the conclusion that ALG-2 interaction with ALIX has a critical role in generating functional ALIX, which supports MVB sorting of activated EGFR.

### The ALG-2–ALIX axis promotes MVB sorting of activated EGFR at early endosomes

**Figure 6:**
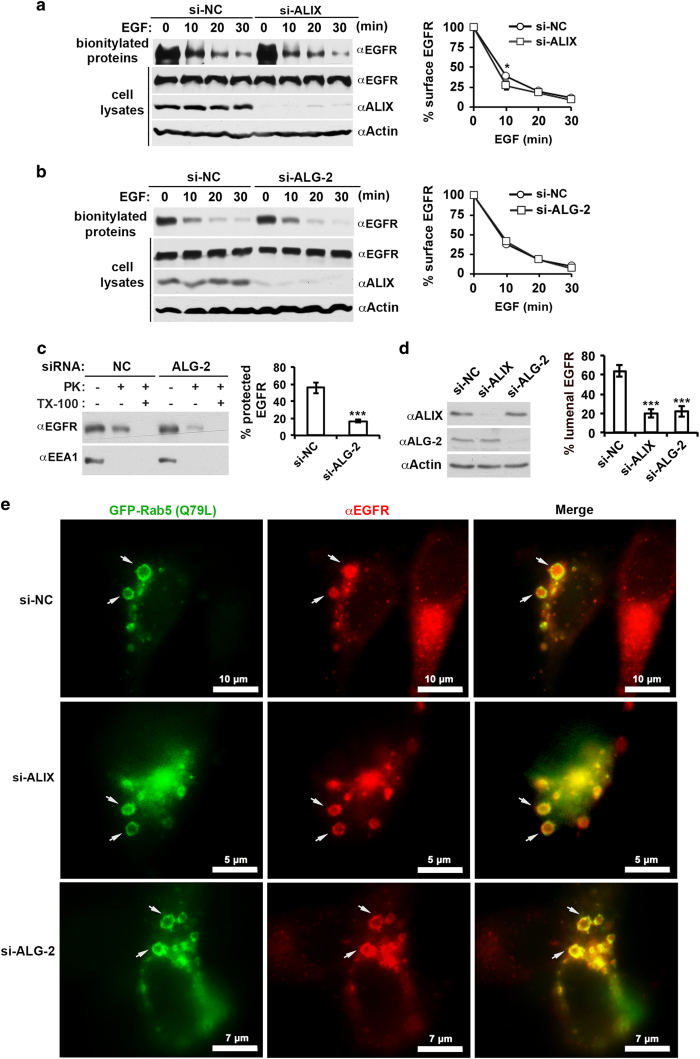
ALG-2 activates the MVB sorting function of ALIX at early endosomes. (**a**) HEK293 cells were transfected with si-NC or si-ALIX(1+2) and cultured for 48 h. After serum starvation for 12 h, cells were stimulated with EGF for indicated minutes and biotinylated. Cell lysates prepared from these cells were incubated with streptavidin beads, and input and bound proteins were immunoblotted with indicated antibodies to visualize EGFR, ALIX, and actin (left panel). The average percentages of cell surface EGFR at different time points and s.d. were determined from three independent experiments and plotted (right panel). (**b**) HEK293 cells were transfected with si-NC or si-ALG-2(1+2) and processed as described in **a** (left panel). The average percentages of cell surface EGFR at different time points and s.d. were determined from three independent experiments and plotted (right panel). (**c**) HEK293 cells were transfected with si-NC or si-ALG-2(1+2) and assayed for EGF-stimulated MVB sorting of EGFR as described in Figure 5a, except that cells were cultured in the presence of 10 μm nocodazole for 2 h before and during EGF stimulation (left panel). The average percentages of protected EGFR and s.d. were determined from three independent experiments and plotted (right panel). (**d**) HeLa cells were first transfected with si-NC, si-ALIX (1+2), or si-ALG-2(1+2) and cultured for 24 h. These cells were then transfected with the expression plasmids for GFP-Rab5 (Q79L) and cultured for another 12 h before serum starvation for 12 h. Lysates of these cells were immunoblotted with indicated antibodies to visualize ALIX, ALG-2, and actin (left panel). These cells were also stimulated with EGF for 30 min, immunostained with an anti-EGFR antibody (red), and observed under a fluorescence microscope. Total and luminal EGFR were quantified for 10 readily discernible GFP-Rab5 (Q79L)-labeled endosomes for each cell condition, and the percentages of luminal EGFR were calculated. The average percentages of luminal EGFR and s.d. were determined from three independent experiments and plotted (right panel). (**e**) Representative images from the experiments described in **d** are shown. Arrows indicate typical GFP-labeled enlarged endosomes with EGFR staining for each cell condition. Note that images of the middle panel and lower panel are magnified for clear visualization of individual endosomes and that scale bars are labeled accordingly. ALG-2, apoptosis-linked gene-2 product; EGF, epidermal growth factor; EGFR, epidermal growth factor receptor; GFP, green fluorescent protein; MVB, multivesicular body; si-NC, negative-control small interfering RNA.

To identify the step(s) at which the ALG-2–ALIX axis promotes MVB sorting of activated EGFR, we first determined the effect of ALIX or ALG-2 knockdown on EGF-induced endocytosis of EGFR, which is a prerequisite for MVB sorting of activated EGFR. Serum-starved HEK293 cells were biotinylated for 30 min upon 0, 10, 20, or 30 min of continuous EGF stimulation, and biotinylated cell surface proteins were incubated with streptavidin beads. EGFR immunoblotting of the absorbed biotinylated proteins showed that ALIX knockdown did not affect the level of cell surface EGFR before EGF stimulation. However, it decreased the cell surface EGFR from 38 to 27% at the 10-min time point, although it had no or little effect afterward (Figure 6a). These results qualitatively concur with the previous results obtained in HeLa and CHO cells [35], and indicate that a certain function of ALIX slightly delays the initial rate of endocytosis of activated EGFR. In contrast to ALIX knockdown, ALG-2 knockdown did not affect the level of cell surface EGFR at any of the four time points examined (Figure 6b), indicating that the inhibitory effect of ALIX on endocytosis of activated EGFR is independent of ALG-2 function. Together, these results eliminate the possibility that promoting endocytosis of activated EGFR has any role in the promoting effect of the ALG-2–ALIX axis on MVB sorting of activated EGFR.

After endocytosis, MVB sorting of activated EGFR may occur at early endosomes or late endosomes. There has been experimental evidence that ALIX moderately inhibits MVB sorting of activated EGFR in cell-free systems at late endosomes [36], presumably through promoting back-fusion of intraluminal vesicles [37]. On the other hand, our previous studies indicated that treating cells with the microtubule poison nocodazole to block early- to late-endosome trafficking [38] did not affect the inhibitory effect of ALIX knockdown on MVB sorting of activated EGFR [9], indicating that ALIX promotes MVB sorting of activated EGFR at early endosomes. To determine whether ALG-2 also promotes MVB sorting of activated EGFR at early endosomes, we treated cells with nocodazole and then determined the effect of ALG-2 knockdown on MVB sorting of activated EGFR. As shown in Figure 6c, nocodazole treatment did not affect the inhibitory effect of ALG-2 knockdown on MVB sorting of activated EGFR. These results indicate that ALG-2 activates the MVB sorting function of ALIX at early endosomes.

To further test our hypothesis by a cell biology approach, we transfected HeLa cells with GFP-tagged Q79L mutant form of small GTPase Rab5 to create enlarged and limiting membrane-labeled early endosomes and determined the effect of ALIX or ALG-2 knockdown on sorting of activated EGFR into these enlarged endosomes by immunofluorescence staining of EGFR. This approach was successfully utilized in multiple previous studies to examine MVB sorting of membrane receptors at early endosomes by using fluorescence microscopy [39, 40]. Note that we switched to HeLa cells for this particular experiment, because HEK293 cells were loosely adherent and thus not suitable for immunostaining and fluorescence microscopy. We had determined that HeLa cells behaved similarly as HEK293 cells in both ligand-dependent MVB sorting of EGFR (Supplementary Figure S5A) and inhibitory effects of ALIX knockdown (Supplementary Figure S5B) or ALG-2 knockdown on MVB sorting of activated EGFR (Supplementary Figure S5C). Quantification of EGFR within GFP-labeled endosomes showed that both ALIX knockdown and ALG-2 knockdown decreased the percentage of intralumenal EGFR from ~60 to ~20% (Figures 6d and e). These results provide more direct evidence that the ALG-2/ALIX axis promotes MVB sorting of activated EGFR at early endosomes.

### Inhibition of ALIX activation by ALG-2 retards degradation of activated EGFR

**Figure 7:**
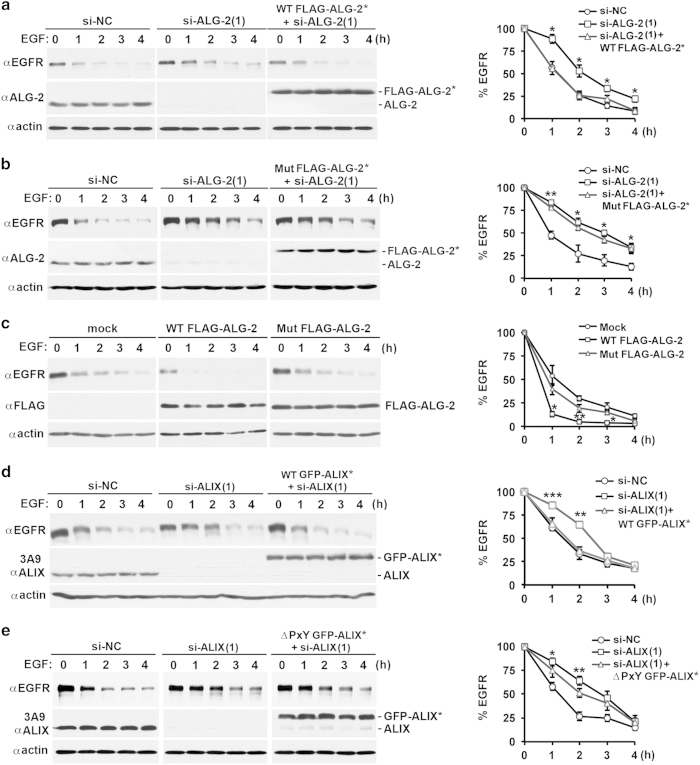
Inhibition of activation of ALIX retards degradation of activated EGFR. (**a, b**) HEK293 cells were transfected with si-NC or si-ALG-2(1) and cultured for 48 h. These cells were then transfected with plasmids for WT FLAG-ALG-2* (**a**) or Mut FLAG-ALG-2* (**b**) and cultured for another 12 h. After serum starvation for 12 h, these cells were stimulated with EGF for the indicated hours, and cell lysates were immunoblotted with indicated antibodies to visualize EGFR, ALG-2, and actin (left panel). The average percentages of remaining EGFR at different time points and s.d. were determined from three independent experiments and plotted (right panel). (**c**) HEK293 cells were transfected with plasmids for WT FLAG-ALG-2 or Mut FLAG-ALG-2 and analyzed for EGF-induced EGFR degradation as described in **a**, except that immunoblotting with an anti-ALG-2 antibody was changed to immunoblotting with an anti-FLAG antibody. (**d, e**) HEK293 cells were transfected with si-NC or si-ALIX(1) and cultured for 48 h. These cells were then transfected with plasmids for WT GFP-ALIX* (**d**) or ∆PxY GFP-ALIX* (**e**) and analyzed for EGF-induced EGFR degradation as described in **a**, except that immunoblotting with an anti-ALG-2 antibody was changed to immunoblotting with the 3A9 anti-ALIX antibody. ALG-2, apoptosis-linked gene-2 product; GFP, green fluorescent protein; EGFR, epidermal growth factor receptor; si-NC, negative-control small interfering RNA; WT, wild type.

ALIX knockdown retards degradation of activated EGFR upon continuous stimulation of serum-starved HEK293 cells with 100 ng ml^−1^ EGF [9] or 20 ng ml^−1^ EGF (Supplementary Figure S6), indicating that ALIX-supported MVB sorting accelerates lysosomal trafficking of activated EGFR to lysosomes. As ALG-2 is responsible for generating functional ALIX that facilitates MVB sorting of activated EGFR, it is conceivable that ALG-2 also accelerates degradation of activated EGFR. To test this prediction, we determined the effect of ALG-2 knockdown or overexpression on the kinetics of EGF-induced EGFR degradation. ALG-2 knockdown moderately elevated the basal level of EGFR (17%, *P*-value<0.05). It also increased the percentage of remaining EGFR at 1, 2, 3, and 4 h. This resulted in retardation of the 50% EGFR degradation from 1 to 2–2.5 h, although the major effect on the rate of decline of remaining EGFR is during the first hour of EGF stimulation. These effects of ALG-2 knockdown were rescued by ectopic expression of WT FLAG-ALG-2*, but not of Mut FLAG-ALG-2* (Figures 7a and b). In contrast to the retardation effect of ALG-2 knockdown, ectopic expression of WT FLAG-ALG-2, but not of Mut FLAG-ALG-2, decreased both the basal level of EGFR (35%, *P*-value<0.05) and the percentage of remaining EGFR at 1, 2, and 3 h, accelerating the 50% EGFR degradation from 1 to 0.5 h (Figure 7c). Again, the major effect on the rate of decline of remaining EGFR is during the first hour of EGF stimulation. These results demonstrate that ALG-2 knockdown retards degradation of activated EGFR. In addition, we determined the effect of the ∆PxY mutation on the ability of GFP-ALIX* to rescue the retardation effect of ALIX knockdown on EGFR degradation. As observed in our previous studies [9], ALIX knockdown had no or little effects on the basal level of EGFR expression, but increased the percentage of remaining EGFR at 1 and 2 h after EGF stimulation. Thus ALIX knockdown was less potent than ALG-2 knockdown in retarding degradation of activated EGFR, which could be due to ALG-2-independent roles of ALIX in inhibiting endocytosis and/or promoting back-fusion at late endosomes [37]. Most importantly, the expression of WT GFP-ALIX* fully rescues the retardation effect of ALIX knockdown on EGFR degradation (Figure 7d), whereas the expression of ∆PxY GFP-ALIX* did not have such a rescuing effect (Figure 7e). These results demonstrate that inhibition of ALG-2 interaction with ALIX retards degradation of activated EGFR.

Together, these results support the prediction that ALG-2-induced activation of the MVB sorting function of ALIX accelerates degradation of activated EGFR under continuous EGF stimulation conditions.

### ALG-2 is not responsible for generating functional ALIX that supports cytokinetic abscission or retroviral budding

**Figure 8:**
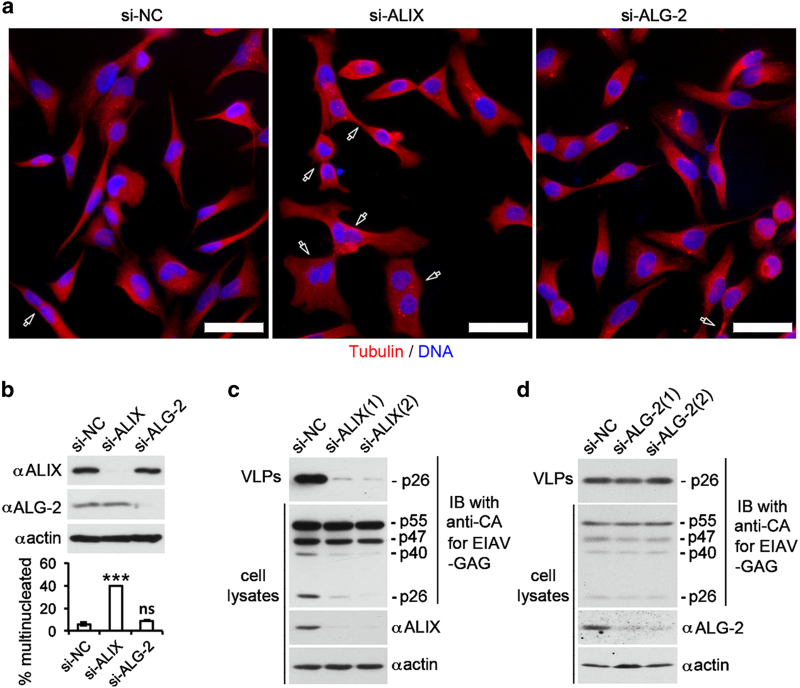
ALG-2 is not responsible for generating functional ALIX that supports cytokinetic abscission or retroviral budding. (**a**, **b**) HeLa cells were transfected with indicated siRNAs and cultured for 72 h. (**a**) After fixation, cells were immunostained with anti-tubulin antibodies (red) and counterstained with the DNA dye DAPI (blue). Representative images are shown, and hollow arrows indicate multinucleated or midbody stage cells. Scale bar: 50 μm. (**b**) Cell lysates were prepared and immunoblotted with indicated antibodies to visualize the expression levels of ALIX and ALG-2 (upper panel). The average percentages of multinucleated cells and s.d. for each cell condition were determined from three independent experiments and plotted (lower panel). (**c**, **d**) HEK293 cells were first transfected with si-NC, si-ALIX(1), or si-ALIX(2) in **c** and with si-NC, si-ALG-2(1), or si-ALG-2(2) in **d** and then transfected with EIAV pEV53B expression vector. Both crude cell lysates and released VLPs were immunoblotted with anti-CA antibodies. Cell lysates were also immunoblotted with the 3A9 anti-ALIX antibody, anti-ALG-2 antibody, and anti-actin antibodies. ALG-2, apoptosis-linked gene-2 product; DAPI, 4ʹ,6-diamidino-2-phenylindole; NS, not significant; si-NC, negative-control small interfering RNA; siRNA, small interfering RNA.

Relieving the intramolecular interaction of ALIX is required for ALIX involvement in all ESCRT-mediated membrane remodeling processes. To determine whether ALG-2 is responsible for generating functional ALIX that supports cytokinetic abscission, we compared the effects of ALIX and ALG-2 knockdown on cytokinetic abscission in HeLa cells. As shown in Figures 8a and b, ALIX knockdown by a combinational use of the two ALIX siRNAs increased the percentage of multinucleated (including the midbody stage) cells from 5 to 35%. In contrast, ALG-2 knockdown by a combinational use of the two ALG-2 siRNAs had no effect or a borderline effect on cytokinetic abscission. These results demonstrate that ALG-2 is not responsible for generating functional ALIX that supports cytokinetic abscission.

To determine whether ALG-2 is responsible for generating functional ALIX that supports retroviral budding, we compared the effects of ALIX knockdown and ALG-2 knockdown on budding of replication-incompetent EIAV. Consistently with previous results by others [13, 41], ALIX knockdown by either of the two siRNAs used markedly inhibited the EIAV budding from infected HEK293 cells (Figure 8c). However, ALG-2 knockdown by either of the two ALG-2 siRNAs did not inhibit EIAV budding (Figure 8d). These results demonstrate that ALG-2 is not responsible for generating functional ALIX that supports retroviral budding.

## Discussion

ESCRT-III-mediated membrane remodeling drives the membrane scission that sorts activated membrane receptors into the lumen of MVBs. Our previous studies demonstrated that CHMP4-bound ALIX at the membrane has a critical role in MVB sorting of activated EGFR [9]. However, ALIX contains a default intramolecular interaction that inhibits ALIX interaction with membrane-bound CHMP4 [20], and the mechanism that relieves the autoinhibition and generates functional ALIX that is capable of supporting MVB sorting of membrane receptors has not been identified. In this study, we demonstrated that ALG-2 interaction with ALIX induces a global conformational change that completely relieves the intramolecular interaction of ALIX. The unlocked ALIX quantitatively moves to the membrane fraction through interaction with membrane-bound CHMP4 and thus primarily performs the membrane-associated ESCRT function. Although this regulatory event is rate-limiting in generating functional ALIX, which supports MVB sorting of activated EGFR at early endosomes, it is not important for ALIX involvement in cytokinetic abscission or retroviral budding. These findings resolve the long-standing issue of the biological function of ALG-2 interaction with ALIX and provide novel insights into the regulation of MVB sorting of activated EGFR and possibly other ubiquitinated membrane receptors.

In ground-state HEK293 cells, only 10% of ALG-2 interacts with ALIX, indicating that the majority of ALG-2 is unable to activate ALIX. If the availability of calcium is the only rate-limiting factor for ALG-2 activation of ALIX, treatment of HEK293 cells with calcium ionophore A23187 or direct addition of calcium to the cytosol should significantly increase the level of activated ALIX. However, addition of calcium to the cytosol or treatment of cells with A23187 either slightly increased or had no effect on the level of activated ALIX. Only in the context of GST-ALG-2 addition or the FLAG-ALG-2 expression, these treatments significantly increased the level of activated ALIX (see Figures 1 and 2). These findings indicate that the majority of ALG-2 in ground-state cells exists in a dormant state that cannot perform calcium-dependent activation of ALIX and predict that protein availability is another rate-limiting factor in the regulation of ALIX activation of ALG-2.

In contrast to a marginal or no effect of calcium alone on ALIX activation by endogenous ALG-2 or FLAG-ALG-2, EGF stimulation of HEK293 cells caused an approximately threefold increase in ALIX activation by endogenous ALG-2 in the absence of increased expression of ALG-2. This predicts that activation of EGFR is able to induce a significant increase in both the calcium concentration and ALG-2 availability. Activation of EGFR is known to link to its endocytosis, and endocytosis is known to induce a calcium spike near endosomes [22, 23]. However, we have yet to understand how activation of EGFR leads to increased ALG-2 availability. Previous studies have demonstrated that ectopic expression of the EGFR-related receptor tyrosine kinase Her2/neu in NIH3T3 cells induces tyrosine phosphorylation of ALG-2 [42]. Raf interacts with and phosphorylates ALG-2 [43]. Thus, it is possible that some EGFR activation-linked post-translational modification of ALG-2 increases ALG-2 availability.

ALG-2 was originally identified by its critical role in apoptotic induction by the T-cell receptor, Fas, and glucocorticoid-induced cell death in T-cell hybridoma [21]. To understand the molecular basis of the pro-apoptotic function of ALG-2, yeast two-hybrid screens were performed to identify ALG-2-interacting proteins. Although these efforts led to the discovery of ALG-2 interaction with ALIX [6, 7] and supportive evidence that this interaction is important for apoptotic induction [21, 44–47], why ALG-2 has a critical role in apoptotic induction remains unresolved. On the other hand, there is abundant evidence that activated growth factor receptors promote cell proliferation and survival through a variety of signaling pathways. Thus, our finding that ALG-2-induced activation of the MVB sorting function of ALIX controls the signaling output of EGFR and possibly many other growth factor receptors generates an interesting hypothesis that ALG-2 positively influences apoptotic induction through controlling the signaling output of activated growth factor receptors.

## Materials and Methods

### Cell culture and transfection

HEK293 and HeLa cells were maintained in Dulbecco’s modified Eagle’s medium (DMEM) (Mediatech, Manassas, VA, USA) supplemented with 2 mm l-glutamine and 10% fetal bovine serum (Atlanta Biologicals, Norcross, GA, USA). Subconfluent cultures of cells in 60-mm or 35-mm culture dishes were transfected with siRNAs or mammalian expression vectors using GenJet DNA *in vitro* transfection reagent or GenMute siRNA transfection reagent (SignaGen Laboratories, Gaithersburg, MD, USA) according to the manufacturers’ instructions. Owing to the high abundance of ALIX, transfection with ALIX-specific siRNAs was done twice (at 0 and 24 h), as performed in multiple previous studies [48, 49]. Transfected cells were cultured for an additional 24–72 h before experimental analyses. siRNAs used in this study are summarized in Supplementary Table S1. Mammalian expression vectors used in this study are summarized in Supplementary Table S2. PCR primers used for site-directed mutagenesis and making vectors are summarized in Supplementary Table S3. Note that transfection efficiency for FLAG-ALG-2 was >90%, as determined by immunostaining transfected cells using anti-FLAG antibodies, and that transfection efficiency for GFP-ALIX was also >90%, as determined by observing transfected cells under a fluorescence microscope.

The calcium ionophore A23187 (Sigma, St Louis, MO, USA) was solubilized in 0.1% dimethyl sulfoxide, and was added to the culture medium at a final concentration of 10 μm 10–15 min before cell collection. To measure EGF-induced EGFR degradation, sub-confluent cultures of cells in 35-mm dishes were first cultured in serum-free medium for 12 h and then cultured in the same medium supplemented with 100 ng ml^−1^ of recombinant EGF (Sigma) for indicated lengths of time. Cell-permeable calcium chelator BAPTA-AM (Toronto Research Chemicals, Toronto, ON, Canada) was solubilized in dimethyl sulfoxide and added to the culture medium at a final concentration of 10 μm 1 h before EGF stimulation. Nocodazole (Sigma) was solubilized in dimethyl sulfoxide and added to the culture medium at a final concentration of 10 μm 2 h before EGF stimulation and remained in the culture medium during the process of stimulation [38].

### Protein extraction and immunoblotting

To prepare crude cell lysates for immunoblotting, cells scraped from culture plates were pelleted and extracted with cell lysis buffer consisting of 50 mm Tris-HCl, 150 mm NaCl, 1% Triton X-100, 0.1% SDS, 0.5 mm EDTA, 100 μm sodium orthovandadate, 100 μm sodium fluoride, 100 μm sodium pyrophosphate, 1 mm dithiothreitol and proteinase inhibitor cocktail (Sigma). One 60-mm dish of cells was extracted with 200 μl of cell lysis buffer. Cell lysates were cleared by centrifugation at 16 000* g* for 10 min at 4 °C. Immunoblotting was performed according to our standard protocols [31]. Relative signals on immunoblots were quantified by analyzing scanned images with NIH ImageJ version 1.41o (Bethesda, MD, USA). Antibodies used in this study are summarized in Supplementary Table S4.

### Activation of cytosolic ALIX by recombinant ALG-2

To prepare cytosolic proteins, pelleted cells were extracted by sonication in 10 volumes of TBS (50 mm Tris-HCl, 150 mm NaCl, pH 7.4) supplemented with 100 μm sodium orthovandadate, 100 μm sodium fluoride, 100 μm sodium pyrophosphate, 1 mm dithiothreitol, and proteinase inhibitor cocktail (Sigma). Cell lysates were cleared by centrifugation at 16 000 *g* for 10 min at 4 °C.

GST and GST-tagged proteins were produced and purified using our standard procedures [31]. One to two micrograms of GST or GST-ALG-2 were added into 100 μl of the cytosolic fraction of cell lysates either alone or together with 1–2 μg of GTS-p6 or GST-p9 whenever indicated. CaCl_2_ was added to the cytosolic fraction at a final concentration of 10 μm to activate ALG-2, as previously described [26]. To reverse the activation of ALG-2, EGTA was added to the sample at a final concentration of 5 mm, as previously described [26]. Immunoprecipitation was performed according to our standard protocols [31].

### Membrane floatation centrifugation

The PNS of HEK293 cell lysates was prepared by resuspending cell pellets in 100 μl of 10% (w/v) sucrose in TE buffer (TBS plus 1 mm EDTA) supplemented with proteinase inhibitor cocktail. Cells were lysed by sonication followed by centrifugation at 1 800 *g* for 5 min at 4 °C. Membrane flotation centrifugation of the PNS through a step sucrose gradient was performed according to published protocols [29, 30], with minor modifications as previously described [19]. The relative abundance of ALIX, ALG-2, and CHMP4 between the membrane and soluble protein fractions was determined by analyzing scanned images with NIH ImageJ version 1.41o, as described previously [20]. For immunoprecipitation, pooled membrane and soluble protein fractions were supplemented with 0.1% Triton X-100, which does not relieve the intramolecular interaction of ALIX [19, 27]. After centrifugation, supernatants were immunoprecipitated according to our standard protocols [31].

### Proteinase K protection assay

Subconfluent cultures of HEK293 cells were cultured in a serum-free medium for ~12 h. Recombinant EGF (Sigma) was added to the culture medium at a final concentration of 100 ng ml^−1^, and cells were cultured further for 30 min. The proteinase K protection assay was performed on these cells exactly as described in our recent studies [9].

### Biotinylation of cell surface proteins and affinity absorption of biotinylated proteins

Biotinylation of cell surface proteins was performed according to a published protocol [50] with minor modifications. Serum-starved HEK293 cells cultured in six-well plates were stimulated with 100 ng ml^−1^ EGF for indicated lengths of time followed by a rinse with ice-cold phosphate-buffered saline (PBS) (pH 7.4) supplemented with 1.5 mm MgCl_2_ and 0.2 mm CaCl_2_. Each well of cells was then incubated twice on a shaker with 0.75 ml of freshly prepared cold sulfo-NHS-SS-biotin solution (bioWORLD) (200 mg ml^−1^) at 4 °C for 15 min each, and quenched by washing with a quenching solution (PBS supplemented with 1.5 mm MgCl_2_, 0.2 mm CaCl_2_, and 100 mm glycine, pH 7.4) and further incubation in this solution at 4 °C for 30 min. Cell lysates were prepared as described above and incubated with Streptavidin Separopore (Agarose) 4B (bioWORLD, Dublin, OH, USA) at 4 °C overnight. Pelleted beads were then washed with TBS supplemented with 1% Triton X-100 five times, and proteins were eluted with SDS sample buffer for immunoblotting.

### Immunostaining and fluorescence microscopy

HeLa cells were transfected with indicated siRNAs twice at 0 and 24 h. To visualize sorting of activated EGFR into early endosomes, these cells were transfected with GFP-Rab5 (Q79L), cultured further for 12 h, and serum starved for 12 h. Serum-starved cells were then stimulated with 100 ng ml^−1^ EGF for 30 min, fixed with 4% (w/v) of paraformaldehyde at room temperature for 20 min, and permeabilized with 0.2% Triton X-100 in PBS followed by blocking with blocking buffer (1% bovine serum albumin, 0.25% horse serum, and 0.2% Triton X-100 in PBS). Blocked cells were first stained with anti-EGFR antibodies in 0.1× blocking buffer at 4 °C overnight and then with Alexa Fluor 568-conjugated secondary antibodies in TBST (0.1% Triton X-100 in TBS) at room temperature for 1 h. Images were acquired using MetaMorph software (7.7.5.0, Nashville, TN, USA) on ZEISS Axioplan2 image system (objective: plan-NEOFLUAR ×100/1.30 oil, Jena, Germany). Total and luminal EGFR fluorescence in 10 readily discernible GFP-Rab5(Q79L)-labeled endosomes were quantified for each cell condition in each experiment using MetaMorph software.

To visualize cytokinesis, transfected HeLa cells were subcultured into chamber slides (Nunc Lab-Tek, Waltham, MA, USA) coated with poly-d-lysine (Cultrex, Gaithersburg, MD, USA) and cultured for 48 h. Cells were fixed, permeabilized, and blocked as described above. Blocked cells were first stained with anti-tubulin antibodies in 0.1× blocking buffer at 4 °C overnight and then with Alexa Fluor 568- and Alexa Fluor 488-conjugated secondary antibodies (Life Technologies, Grand Island, NY, USA) in TBST (0.1% Triton X-100 in TBS) at room temperature for 1 h. Finally, nuclei were counterstained with 4ʹ,6-diamidino-2-phenylindole (Sigma), and images were acquired using MetaMorph software (7.7.5.0) on a ZEISS Axioplan2 image system (objective: plan-NEOFLUAR ×20/0.50). For obtaining the percentages of multinucleated cells induced by ALIX knockdown, at least 200 cells were counted for each experiment.

### EIAV VLP release assay

The EIAV virus-like particle (VLP) release assay was performed according to published protocols [13]. In brief, HEK293 cells were transfected with pEV53B EIAV vector [51] and cultured for 48 h. Conditioned medium was then collected and loaded onto a 2-ml 20% sucrose cushion in a 4-ml tube. After ultracentrifugation of the sample in an SW55-Ti rotor at 26 000 r.p.m. for 2 h, pelleted proteins were immunoblotted with anti-EIAV capsid antigen antibodies.

### Statistical analysis

Statistical analyses were performed using the Student’s *t*-test. *P-values ⩾0.01 and <0.05 were considered significant. The **P-values ⩾0.001 and <0.01 were considered highly significant. A ***P-value of <0.001 was considered very highly significant.
