# ALG-2 activates the MVB sorting function of ALIX through relieving its intramolecular interaction

**DOI:** 10.1038/celldisc.2016.23

**Published:** 2016-06-21

**Authors:** Sheng Sun, Xi Zhou, Joe Corvera, Gary E Gallick, Sue-Hwa Lin, Jian Kuang

**Affiliations:** 1 Department of Experimental Therapeutics, The University of Texas MD Anderson Cancer Center, Houston, TX, USA; 2 The University of Texas Graduate School of Biomedical Sciences, Houston, TX, USA; 3 A&G Pharmaceuticals, Inc., Baltimore, MD, USA; 4 Department of Genitourinary Medical Oncology, The University of Texas MD Anderson Cancer Center, Houston, TX, USA; 5 Department of Molecular Pathology, The University of Texas MD Anderson Cancer Center, Houston, TX, USA

**Correction to:** *Cell Discovery* (2015) **1**, 15018; doi:10.1038/celldisc.2015.18; published online 21 July 2015

In the initial published version of this article, there was an error in the Supplementary Information linking description on page 19. “Supplementary Information is linked to the online version of the paper on the *Cell Research* website.” should be changed into “Supplementary Information is linked to the online version of the paper on the *Cell Discovery* website.”

We apologize for any inconvenience that may have been caused by this error.

