# Supplementary material for: ALG-2 activates the MVB sorting function of ALIX through relieving its intramolecular interaction
Source: Cell Discov. 2015 Jul 21;1:15018–. doi: 10.1038/celldisc.2015.18 (PMC4860835; doi:10.1038/celldisc.2015.18)
Supplement: Supplementary Information [file celldisc201518-s1.pdf]

SUPPLEMENTAL MATERIALS AND METHODS

Co-immunoprecipitation

To prepare cell lysates for co-immunoprecipitation of ALG-2 with ALIX in control and EGF-stimulated cells, pelleted cells were extracted with sonication in 10 volumes of TBS supplemented with 0.1% Triton X-100, 100 μM sodium orthovandadate, 100 μM sodium fluoride, 100 μM sodium pyrophosphate, 1 mM dithiothreitol (DTT) and proteinase inhibitor cocktail (Sigma). 0.1% Triton X-100 was added to solubilize membrane. After cell lysates were cleared by centrifugation at 16,000 *g* for 10 min at 4°C, samples were supplemented with 10 μM CaCl2 and immunoprecipitated with mouse IgG or the 3A9 anti-ALIX antibody.

GST pull-down assay

GST and GST tagged proteins were produced and purified using our standard procedures and immobilized onto glutathione beads (GenScript). Cytosolic proteins prepared from HEK293 cells (~100 μl) were incubated with 1-2 μg immobilized GST or GST-ALG-2 in the presence or absence of 10 μM $CaCl_2$ or 5 mM EGTA at 4ºC for 2 h. After beads were washed with TBS supplemented with 1% Triton X-100 five times, bound proteins were eluted with SDS sample buffer for immunoblotting.

Table S1. Sequences of siRNAs used in this study

Target	Name	Sequence	Source
ALIX	si-ALIX(1)	5’-GAGAAGAAAUUGCAAGGUUdTdT-3’	Sigma-Genosys
ALIX	si-ALIX(2)	5’-GAAGGAUGCUUUCGAUAAAdTdT-3’	Sigma-Genosys
ALG-2	si-ALG-2(1)	5’-GGUCGAUCAUAUCCAUGUUdTdT-3’	Sigma-Genosys
ALG-2	si-ALG-2(2)	5’-GACAGGAGUGGAGUGAUAUdTdT-3’	Sigma-Genosys
Firefly GL3 luciferase	si-NC	5’-CUUACGCUGAGUACUUCGAdTdT-3’	Sigma-Genosys
CHMP4B	si-CHMP4B(1)	5’-AGAAGAGUUUGACGAGGAUdTdT-3’	Sigma-Genosys
CHMP4B	si-CHMP4B(2)	5’-CGGAAGAGAUGUUAACGAAdTdT-3’	Sigma-Genosys

Table S2. Mammalian and bacterial expression vectors used in this study

Vector	Source	Reference
1. pEGFP-C3-based mammalian expression vector for GFP-ALIX	A gift from Dr. Masatoshi Maki (Nagoya, Japan)	(1)
1a. pEGFP-C3-based expression vector for ALIX-siRNA(1)-insensitive GFP-ALIX (GFP-ALIX*)	Generated in our previous studies	(2)
1b. pEGFP-C3-based expression vector for I212D GFP-ALIX	Generated in our previous studies	(2)
1c. pEGFP-C3-based mammalian expression vector for ΔPxY GFP-ALIX	A gift from Dr. Masatoshi Maki (Nagoya, Japan)	(1)
1d. pEGFP-C3-based mammalian expression vector for ΔPxY GFP-ALIX*	Site-directed mutagenesis of vector 1c	new
2. pCMV-Tag2C-based mammalian expression vector for FLAG-ALG-2	A gift from Dr. Changmin Chen (Boston, MA)	(3)
2a. pCMV-Tag2C-based mammalian expression vector for ALG-2-siRNA(1)-insensitive FLAG-ALG-2 (FLAG-ALG-2*)	Site-directed mutagenesis of vector 2	new
2b. pCMV-Tag2C-based mammalian expression vector for E47A FLAG-ALG-2	Site-directed mutagenesis of vector 2	new
2c. pCMV-Tag2C-based mammalian expression vector for E47A/E114A FLAG-ALG-2	Site-directed mutagenesis of vector 2b	new
2d. pCMV-Tag2C-based mammalian expression vector for E47A/E114A FLAG-ALG-2*	Site-directed mutagenesis of vector 2c	new
3. pCMV-based mammalian expression vector for FLAG-CHMP4b	A gift from Dr. Masatoshi Maki (Nagoya, Japan)	(4)
4. The pIRES2-based mammalian expression vector for FLAG–TSG101	A gift from Dr. Wesley I. Sundquist (Salt Lake City, UT)	(5)
5. pEGFP-C3-based mammalian expression vector for GFP-Rab5 Q79L	A gift from Jean Gruenberg (Geneva, Switzerland)	(6)
6. pEV53B-based mammalian expression vector for infection defective EIAV	A gift from Dr. John Olsen (Chapel Hill, NC)	(7)
7. pGEX-4T3 based bacterial expression vector for WT GST-ALG-2	PCR amplification of the coding region for ALG-2 from vector 2, followed by subcloning into pGEX-4T3 vector (Amersham Biosciences)	new
8. pGEX-4T3 based bacterial expression vector for E47A/E114A GST-ALG-2	PCR amplification of the coding region for E47A/E114A ALG-2 vector 2C, followed by subcloning into pGEX-4T3 vector	new
9. pGEX-4T3 based bacterial expression vector for GST-$p6^{HIV\text{-}Gag}$	A gift from Dr. Wesley I. Sundquist (Salt Lake City, UT)	(8)
10. pGEX-4T3 based vector bacterial expression for GST-$p9^{EIAV\text{-}Gag}$	A gift from Dr. Wesley I. Sundquist (Salt Lake City, UT)	(8)

REFERENCES

(1) Shibata H, Yamada K, Mizuno T, Yorikawa C, Takahashi H, Satoh H, Kitaura Y, Maki M. The penta-EF-hand protein ALG-2 interacts with a region containing PxY repeats in Alix/AIP1, which is required for the subcellular punctate distribution of the amino-terminal truncation form of Alix/AIP1. J Biochem. 2004 Jan; 135(1):117-28.

(2) Sun S, Zhou X, Zhang W, Gallick GE, Kuang J. Unraveling the pivotal role of ALIX in MVB sorting and silencing of activated EGFR. Biochem J. 2014 Dec 16. [Epub ahead of print]

(3) Chen C, Sytkowski AJ. Apoptosis-linked gene-2 connects the Raf-1 and ASK1 signalings. Biochem Biophys Res Commun. 2005 Jul 22; 333(1):51-7.

(4) Katoh K, Shibata H, Suzuki H, Nara A, Ishidoh K, Kominami E, Yoshimori T, Maki M. The ALG-2-interacting protein Alix associates with CHMP4b, a human homologue of yeast Snf7 that is involved in multivesicular body sorting. J Biol Chem. 2003 Oct 3; 278(40):39104-13. Epub 2003 Jul 14.

(5) von Schwedler UK, Stuchell M, Müller B, Ward DM, Chung HY, Morita E, Wang HE, Davis T, He GP, Cimbora DM, Scott A, Kräusslich HG, Kaplan J, Morham SG, Sundquist WI. The protein network of HIV budding. Cell. 2003 Sep 19; 114(6):701-13.

(6) Brankatschk B, Wichert SP, Johnson SD, Schaad O, Rossner MJ, Gruenberg J. Regulation of the EGF transcriptional response by endocytic sorting. Sci Signal. 2012 Mar 13;5(215):ra21.

(7) Olsen JC. Gene transfer vectors derived from equine infectious anemia virus. Gene Ther. 1998 Nov; 5(11):1481-7.

(8) Fisher RD, Chung HY, Zhai Q, Robinson H, Sundquist WI, Hill CP. Structural and biochemical studies of ALIX/AIP1 and its role in retrovirus budding. Cell. 2007 Mar 9; 128(5):841-52.

Table S3. PCR primers used for site-directed mutagenesis and making vectors

Product vector	Primers (Forward/Reverse)	Template vector
1d. ΔPxY GFP-ALIX*	5'-GAAGAAATTTGGAGAGGAAATTGCAAGGTTAC-3'<br>5'-GTAACCTTGCAATTTCCTCTCCAAATTTCTTC-3'	1c. ΔPxY GFP-ALIX
2a. WT FLAG-ALG-2*	5'-GTGACTGTCAGGTCCATCATATCCATGTTTG-3'<br>5'-CAAACATGGATATGATGGACCTGACAGTCAC-3'	2. WT FLAG-ALG-2
2b. E47A FLAG-ALG-2	5'-GAGTGATATCAGACACCGCGCTTCAGCAAGCTCTCTC-3'<br>5'-GAGAGAGCTTGCTGAAGCGCGGTGTCTGATATCACTC-3'	2. WT FLAG-ALG-2
2c. E47A/E114A FLAG-ALG-2	5'-GATGATCGATAAGAACGCGCTGAAGCAGGCCCTCTCAG-3'<br>5'-CTGAGAGGGCCTGCTTCAGCGCGTTCTTATCGATCATC-3'	2b. E47A FLAG-ALG-2
2d. E47A/E114A FLAG-ALG-2*	5'-GTGACTGTCAGGTCCATCATATCCATGTTTG-3'<br>5'-CAAACATGGATATGATGGACCTGACAGTCAC-3'	2c. E47A/E114A FLAG-ALG-2
6. WT GST-ALG-2	5'-TAAGAATTCCATGGCCGCCTACTCTTAC-3' (EcoR1)<br>5'-TAACTCGAGTCATACGATACTGAAGACCATG-3' (Xho1)	2. WT FLAG-ALG-2
7. E47A/E114A GST-ALG-2	5'-TAAGAATTCCATGGCCGCCTACTCTTAC-3' (EcoR1)<br>5'-TAACTCGAGTCATACGATACTGAAGACCATG-3' (Xho1)	2c. E47A/E114A FLAG-ALG-2

Table S4. Antibodies used in this study

Antibody	Recognition	Type	Source	Use
1A3 anti-ALIX	ALIX (Y319)/ Xp95(Y318)	Mouse monoclonal	Made in our previous studies (1)	Immunoprecipitation
1A12 anti-ALIX	$ALIX_{605-709}$	Mouse monoclonal	Made in our previous studies (1)	Immunoprecipitation
1F7 anti-ALIX	$ALIX_{436-709}$	Mouse monoclonal	Made in our previous studies (1)	Immunoprecipitation
2H12 anti-ALIX	$ALIX_{F676\ pocket}$	Mouse monoclonal	Made in our previous studies (1)	Immunoprecipitation
3A9 anti-ALIX	$ALIX_{605-709}$	Mouse monoclonal	Made in our previous studies (1)	Immunoblotting Immunoprecipitation
anti-actin	Actin	Mouse monoclonal	Sigma-Aldrich Cat#: A5441	Immunoblotting
Anti-ALG-2	ALG-2	Rabbit monoclonal	Epitomics Cat#: 3846-1	Immunoblotting
anti-tubulin	α-tubulin	Rabbit monoclonal	Cell Signaling Cat#: 2125S	Immuno-fluorescence staining
anti-CHMP4B	CHMP4B	Rabbit polyclonal	Santa Cruz Cat#: sc-134946	Immunoblotting
*anti-CHMP4A	CHMP4A	Rabbit polyclonal	Santa Cruz Cat#: sc-67229	Immunoblotting
*anti-CHMP4B/C	CHMP4B/C	Rabbit polyclonal	Abcam Cat#: ab76334-100	Immunoblotting
anti-CA	EIAV capsid antigen (CA)	Mouse monoclonal	A gift from Dr. Robert Mealey (Pullman, WA) (2,3)	Immunoblotting
anti-EEA1	EEA1	Rabbit monoclonal	Epitomics Cat#: 3704-1	Immunoblotting
anti-EGFR	EGFR	Rabbit monoclonal	Epitomics Cat#: 1902-1	Immunoblotting Immuno-fluorescence staining
anti-ERK1	ERK1	Rabbit polyclonal	Santa Cruz Cat#:sc-94	Immunoblotting
anti-ERK2	ERK2	Rabbit polyclonal	Santa Cruz Cat#:sc-154	Immunoblotting
anti-FLAG	FLAG epitope	Mouse monoclonal	Pierce Cat#: MA1-918781	Immunoblotting Immunoprecipitation
anti-GFP	GFP	Mouse monoclonal	Santa Cruz Cat#: sc-9996	Immunoblotting
anti-GST	GST	Rabbit polyclonal	Santa Cruz Cat#: sc-459	Immunoblotting
IgG	IgG	Mouse	Sigma-Aldrich Cat#: I5381-10MG	Immunoprecipitation
anti-p-ERK	p-ERK1/2 at Tyr 204	Mouse monoclonal	Santa Cruz Cat#: sc7383	Immunoblotting

*These two antibodies are used together in immunoblotting of total CHMP4.

REFERENCES

(1) Pan S, Wang R, Zhou X, He G, Koomen J, Kobayashi R, Sun L, Corvera J, Gallick GE, Kuang J. Involvement of the conserved adaptor protein Alix in actin cytoskeleton assembly. J Biol Chem. 2006 Nov 10; 281(45):34640-50. Epub 2006 Sep 10.

(2) McGuire, T. C., O'Rourke, K. I., Baszler, T. V., Leib, S. R., Brassfield, A. L. and Davis, W. C. (1994) Expression of functional protease and subviral particles by vaccinia virus containing equine infectious anaemia virus gag and 5' pol genes. J. Gen. Virol. 75, 895–900

(3) Mealey, R. H., Leib, S. R., Littke, M. H., Wagner, B., Horohov, D. W. and McGuire, T. C. (2009) Viral load and clinical disease enhancement associated with a lentivirus cytotoxic T lymphocyte vaccine regimen. Vaccine 27, 2453–2468

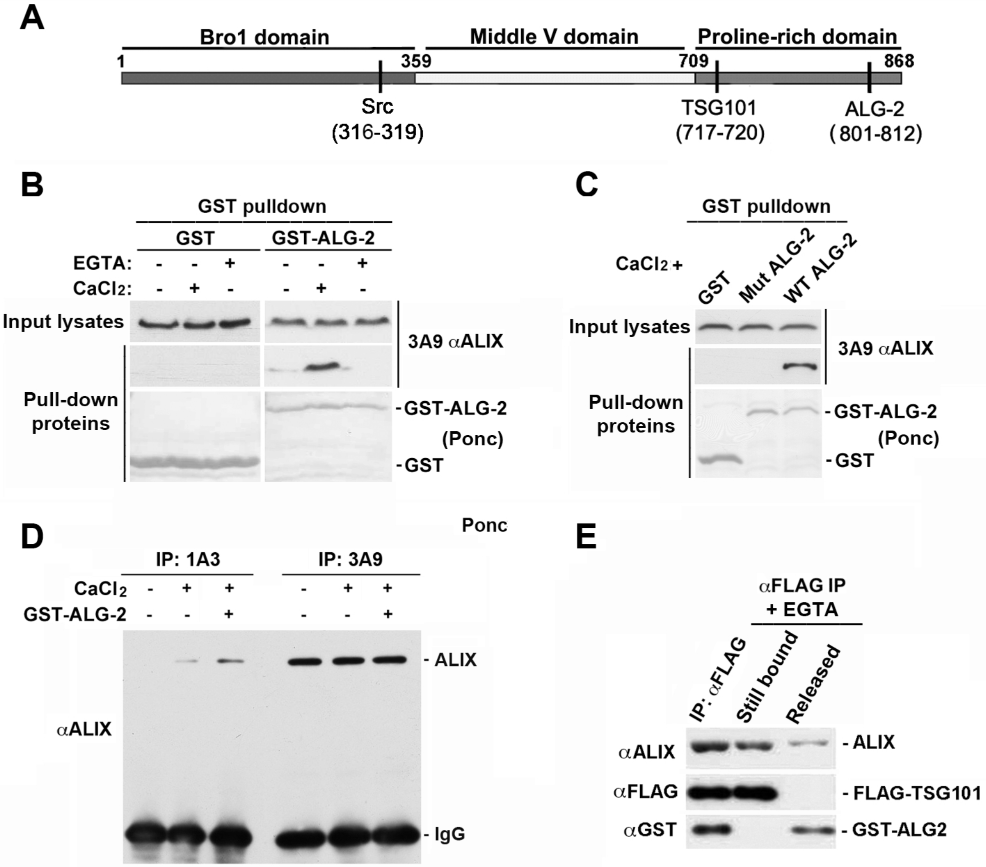

Figure S1. Supplemental data for Fig. 1. (A) Schematic illustration of human ALIX domains and locations of the docking sites for Src, TSG101 and ALG-2. (B) Immobilized GST, WT GST-ALG-2 or Mut GST-ALG-2 was incubated with the cytosolic fraction of HEK293 cell lysates supplemented with nothing, $CaCl_2$ or EGTA. Input cell lysates were immunoblotted with the 3A9 anti-ALIX antibody, and bound proteins were immunoblotted with the 3A9 anti-ALIX antibody after Ponceau S (Ponc) staining. (C) Immobilized GST, WT GST-ALG-2 or Mut GST-ALG-2 was incubated with the cytosolic fraction of HEK293 cell lysates supplemented with $CaCl_2$. Input cell lysates were immunoblotted with the 3A9 anti-ALIX antibody, and bound proteins were immunoblotted with the 3A9 anti-ALIX antibody after Ponceau S (Ponc) staining. (D) Cytosolic proteins from HEK293 cells were mixed with nothing, $CaCl_2$, or $CaCl_2$ plus GST-ALG-2 and immunoprecipitated with the 1A3 or 3A9 anti-ALIX antibody. Immunocomplexes were immunoblotted with anti-ALIX antibodies to visualize ALIX and IgG. (E) The cytosolic fraction of HEK293 cells ectopically expressing FLAG-TSG101 was mixed with GST-ALG-2 plus $CaCl_2$ and immunoprecipitated with an anti-FLAG antibody. The FLAG-TSG101/ALIX/GST-ALG-2 immunocomplex was treated with EGTA, and both released proteins and remaining proteins on the beads were recovered. Proteins in the input sample, in the supernatant (released) and on the beads (bound) were immunoblotted with indicated antibodies to visualize ALIX, FLAG-TSG101 and GST-ALG-2.

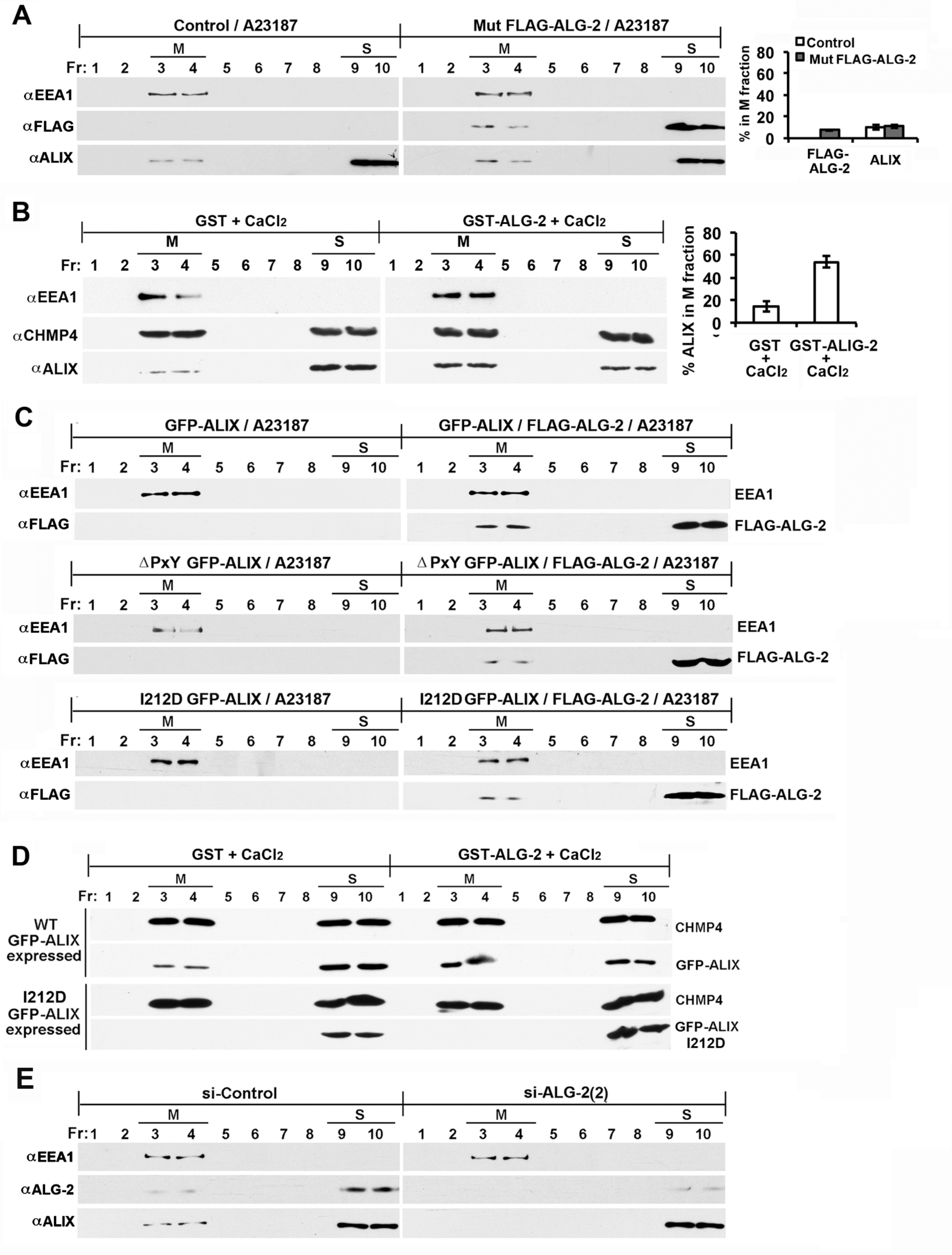

Figure S2. Supplemental data for Fig. 2. (A) HEK293 cells were transfected with an empty vector (control) or an expression vector for Mut FLAG-ALG-2, and processed as described for Fig. 2A (left panel). The average percentages of ALIX and FLAG-ALG-2 in the M fraction were determined from two independent experiments and plotted; error bars indicate the range of the results (right panel). (B) The PNS of HEK293 cells was mixed with $CaCl_2$ plus GST or $CaCl_2$ plus GST-ALG-2 and fractionated by membrane flotation centrifugation. Proteins in collected fractions were immunoblotted with indicated antibodies to visualize EEA1, CHMP4, and ALIX (left panel). The average percentages of ALIX in the M fraction were determined from two independent experiments and plotted; error bars indicate the range of the results (right panel). (C) These immunoblots of EEA1 and FLAG-ALG-2 are part of the experiments described for Fig. 2D. (D) The PNS of HEK293 cells ectopically expressing WT or I212D GFP-ALIX was processed as described for (B), except that proteins in collected fractions were immunoblotted with indicated antibodies to visualize CHMP4 and GFP-ALIX. (E) These immunoblots are part of the experiments described for Fig. 2E.

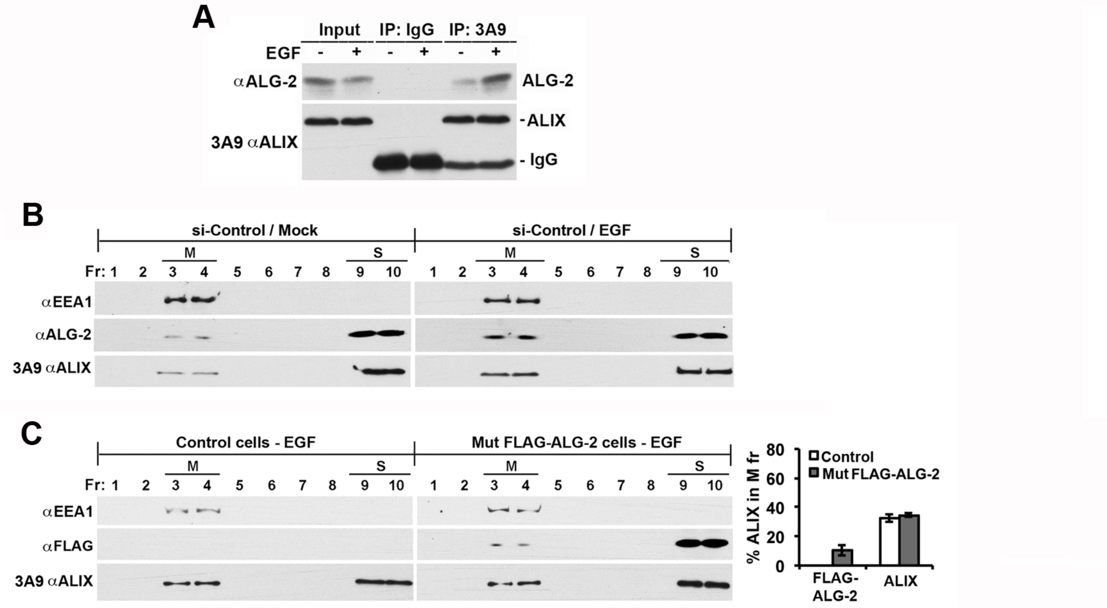

Figure S3. Supplemental data for Fig. 3. (A) HEK293 cells were mock-treated or stimulated with EGF for 1 h, and cell lysates were prepared as described for preparation of cytosolic proteins except that 0.1% Triton X-100 was added to the extraction buffer. These cell lysates were mixed with $CaCl_2$, and then immunoprecipitated with the 3A9 anti-ALIX antibodies. Input proteins and immunocomplexes were immunoblotted with indicated antibodies to visualize ALG-2, ALIX and IgG. (B) These immunoblot results are part of the experiments described for Fig. 3C. (C) HEK293 cells were transfected with an empty vector (control) or an expression vector for Mut FLAG-ALG-2, and processed as described for Fig. 3D (left panel). The average percentages of ALIX and FLAG-ALG-2 in the M fraction were determined from two independent experiments and plotted; error bars indicate the range of the results (right panel).

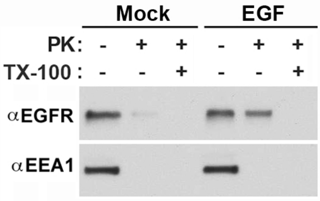

Figure S4. Supplemental data for Fig. 5. HEK 293 cells were serum starved for 12 h and then mock-treated or stimulated with EGF for 30 min. These cells were assayed for MVB sorting of activated EGFR by the proteinase K protection assay.

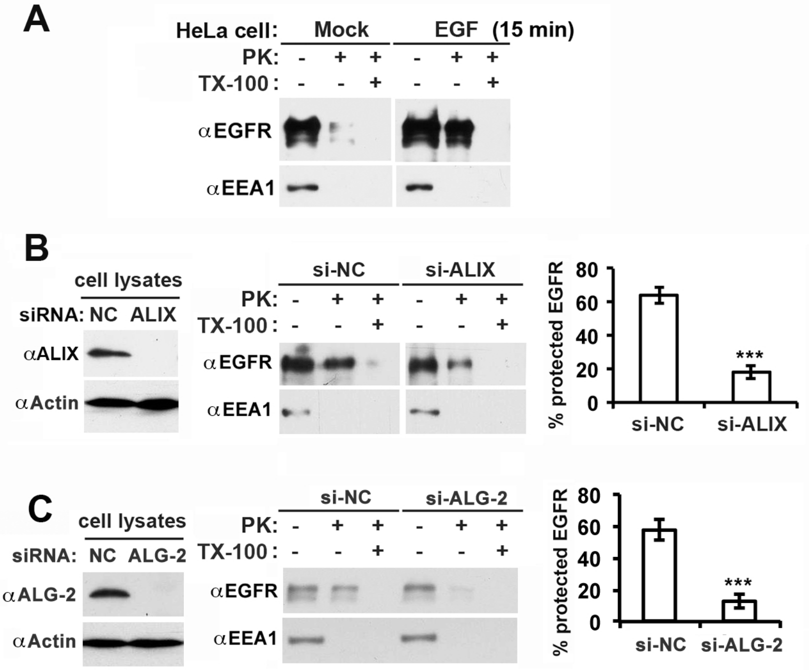

Figure S5. Supplemental data for Fig. 6. (A) HeLa cells were serum starved for 12 h and then mock-treated or stimulated with EGF. These cells were assayed for MVB sorting of activated EGFR by the proteinase K protection assay. (B&C) HeLa cells were transfected with si-NC or si-ALIX(1+2) in (B) and with si-NC or si-ALG-2(1+2) in (C) and cultured for 48 h. After serum starvation for 12 h, these cells were stimulated with EGF and assayed for MVB sorting of activated EGFR by proteinase K protection assay (left panel). The average percentages of protected EGFR and SDs were determined from three independent experiments and plotted (right panel).

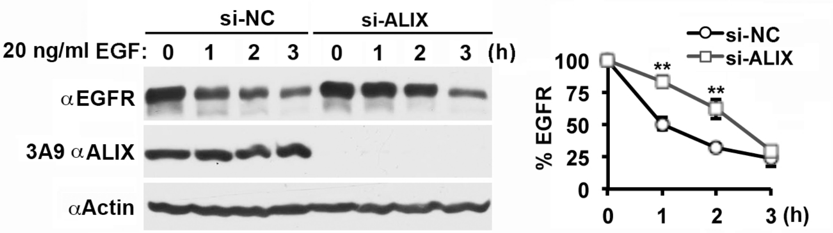

Figure S6. Supplemental data for Fig. 7. HEK293 cells were transfected with si-NC or si-ALIX(1+2) and cultured for 48 h. After serum starvation for 12 h, these cells were stimulated with 20 ng/mL EGF for indicated hours, and cell lysates were immunoblotted with indicated antibodies to visualize EGFR, ALIX and actin (left panel). The average percentages of remaining EGFR at different time points and SDs were determined from three independent experiments and plotted (right panel).
